# Assessment of apical extrusion in regenerative endodontics: a comparative study of different irrigation methods using three-dimensional immature tooth models

**DOI:** 10.1007/s10266-024-00961-x

**Published:** 2024-06-24

**Authors:** Vahide Hazal Abat, Gökçen Deniz Bayrak, Mustafa Gündoğar

**Affiliations:** 1 Department of Endodontics, Faculty of Dentistry, Uskudar University, Istanbul, Turkey; 2 Department of Pediatric Dentistry, Faculty of Dentistry, Yeditepe University, Istanbul, Turkey; 3 Department of Endodontics, Faculty of Dentistry, Medipol University, Istanbul, Turkey

**Keywords:** Apical extrusion, Immature teeth, Irrigation, Laser, Regenerative endodontics, SWEEPS

## Abstract

To investigate the apical extrusion of sodium hypochlorite in immature permanent teeth caused by conventional syringe irrigation (CSI), EDDY, XP-endo Finisher file (XP-F), and a new laser irrigation activation system [shock-wave-enhanced-emission-photo-acoustic-streaming (SWEEPS)]. Three-dimensionally printed forty-nine teeth with immature central incisor morphology were randomly assigned to seven experimental groups, based on the irrigation activation methods and insertion depths (1 mm and 2 mm short of the working length) as follows: CSI-1, CSI-2, EDDY-1, EDDY-2, XP-1, XP-2, and SWEEPS. Prior to the irrigation activation process, samples were placed in metacresol mixed agar gel in Eppendorf tubes. To evaluate NaOCI extrusion into the gel, each sample was digitally photographed, and the area of apical extrusion was analyzed using ImageJ software. To examine potential significant differences between the continuous variables, the Mann–Whitney U test and Kruskal–Wallis H test, were applied (*P* = .05). The SWEEPS resulted in a greater amount of apical extrusion compared to the CSI method, regardless of the insertion depth (*P* < 0.001). The SWEEPS resulted in greater apical extrusion scores compared to EDDY-2 (*P* < 0.001). The EDDY-1 resulted in greater amount of apical extrusion scores compared to EDDY-2 (*P* < 0.001). This study, the first to show the effect of the novel SWEEPS technology on NaOCI extrusion, found that irrigation activation can cause different levels of apical extrusion depending on the method and distance from the working length. It is crucial to consider the potential occurrence of apical extrusion when applying activation methods to immature teeth.

## Introduction

In today’s context, regenerative endodontic treatment (RET) is one of the frequently preferred treatment options for immature permanent teeth with pulp necrosis or apical lesions [1]. However, performing mechanical instrumentation during RET in immature permanent teeth has always been a challenge due to the risk of further weakening the dentinal walls of the canal [2]. At this point, disinfection of the root canal through irrigation and intracanal medication plays a pivotal role in the success of RET, because infection negatively affects regeneration and stem cell activity [3, 4].

Sodium hypochlorite (NaOCl) is the most frequently utilized irrigant in endodontic treatment [5, 6]. It is regarded as the primary endodontic irrigant owing to its antimicrobial efficacy and its capability to dissolve soft tissues within the root canal [6]. The conventional syringe irrigation (CSI) approach is widely used in clinical practice [7]; however, its ability to effectively irrigate can be compromised by the complicated three-dimensional microstructure of the root canal system [8, 9]. Various irrigation activation techniques have been developed to enhance the cleaning abilities of NaOCI.

EDDY (VDW, Munich, Germany), a sonic irrigation activation method, contains a polyamide tip that moves in a three-dimensional manner at high amplitude. This three-dimensional movement removes of debris from hard-to-reach areas in the root canal by triggering acoustic streaming, which increases cleaning efficiency [10].

The XP-Endo Finisher (XP-F) (FKG Dentaire, LaChaux-de-Fonds, Switzerland) is a new rotary nickel-titanium (NiTi) instrument made of a proprietary alloy (MaxWire; Martensite-Austenite Electropolish Flex, FKG Dentaire) that utilizes shape-memory principles [11]. The XP-Endo is a size #25 non-tapered instrument designed to adapt to the original morphology of the root canal and facilitate thorough cleaning of irregular areas due to its increased flexibility. It maintains a straight form in the martensitic phase at room temperature and a curved shape in the austenitic phase at body temperature [12], assuming a spoon shape of 1.5 mm depth in the last 10 mm of its length [11]. According to the manufacturer, the austenitic phase shape enables the files to clean hard-to-reach areas that may be inaccessible to other instruments, when inserted into the canal in rotation mode [13]. This allows the instrument to withstand cyclic fatigue caused by the alternating tension–compression cycles that NiTi files experience when bending in the area of greatest curvature of the canal [14]. The cross-sectional design of this alloy has a larger cross-sectional area and mass compared to the S-shaped instruments [15]. As a result, it has an increased polar moment of inertia, because its mass is dispersed further from the pivot center [15]. An increased polar moment of inertia increases the torsional resistance of the file [16]. The design characteristics of this alloy offer the remarkable advantage of enabling the use of minimally invasive instruments in conjunction with effective cleaning by adapting to different root canal morphologies.

The novel Shock Wave-Enhanced Emission of Photoacoustic Streaming (SWEEPS) mode of laser-activated irrigation (LAI) is based on the interaction between Erbium:Yttrium–Aluminium–Garnet laser (Er:YAG) (wavelength: 2940 nm) and irrigant. This technique differs from previous LAI protocols by emitting a pair of ultrashort pulses of sub-ablative energies (20 mJ). The second laser pulse is applied shortly prior to the collapse of the first pulse's bubble, which results in a high peak pulse power (800 W) and amplification of secondary cavitation that extends to distant areas of the root canal [17, 18]. Furthermore, shock waves created along the canal walls continue to move at supersonic rates as they reach the smear layer, which could improve the cleaning efficiency [17].

Although irrigation activation methods offer efficient root canal cleaning, they might cause irritation of the healthy apical tissue when the irrigation solution is extruded, particularly in immature teeth with wide-open apex. The undesirable extrusion of irrigation solution into periapical tissues raises concerns for RET. Therefore, the preservation of vitality and proliferative potential of stem cells in periapical tissues is crucial for the success of RET [19, 20].

Literature supports irrigation activation for efficient cleanliness, but its application in regenerative endodontics raises concerns due to apical extrusion risk [9, 19, 20]. To address this, apical extrusion studies on immature teeth are crucial. However, there is a lack of research on apical extrusion caused by these irrigation technologies, in teeth with open apices. Even, there is no study employing the novel SWEEPS irrigation method in immature teeth which was utilized in our investigation. This study offers valuable insights into novel irrigation activation in immature teeth. Accordingly, the study aimed to evaluate the apical extrusion of sodium hypochlorite following various irrigation methods, including CSI, EDDY, XP-endo Finisher file, and SWEEPS activation, in immature teeth. The first null hypothesis stated that there is a significantly difference in the amount of apical extrusion generated by each irrigation method when used at distances of 1 mm and 2 mm. The second null hypothesis is that there would be no significant difference in terms of apical extrusion of NaOCI among the various activation methods used.

## Materials and methods

This study did not require ethical approval as it was conducted on models produced by a 3D printer. A power calculation was performed G*Power software (version 3.1, Heinrich Heine University, Dusseldorf, Germany), and the minimum sample size for each group was determined at 7 considering 5% confidence interval with a power of 90%. A total of 49 specimens were manufactured for the present study.

**Fig. 1:**
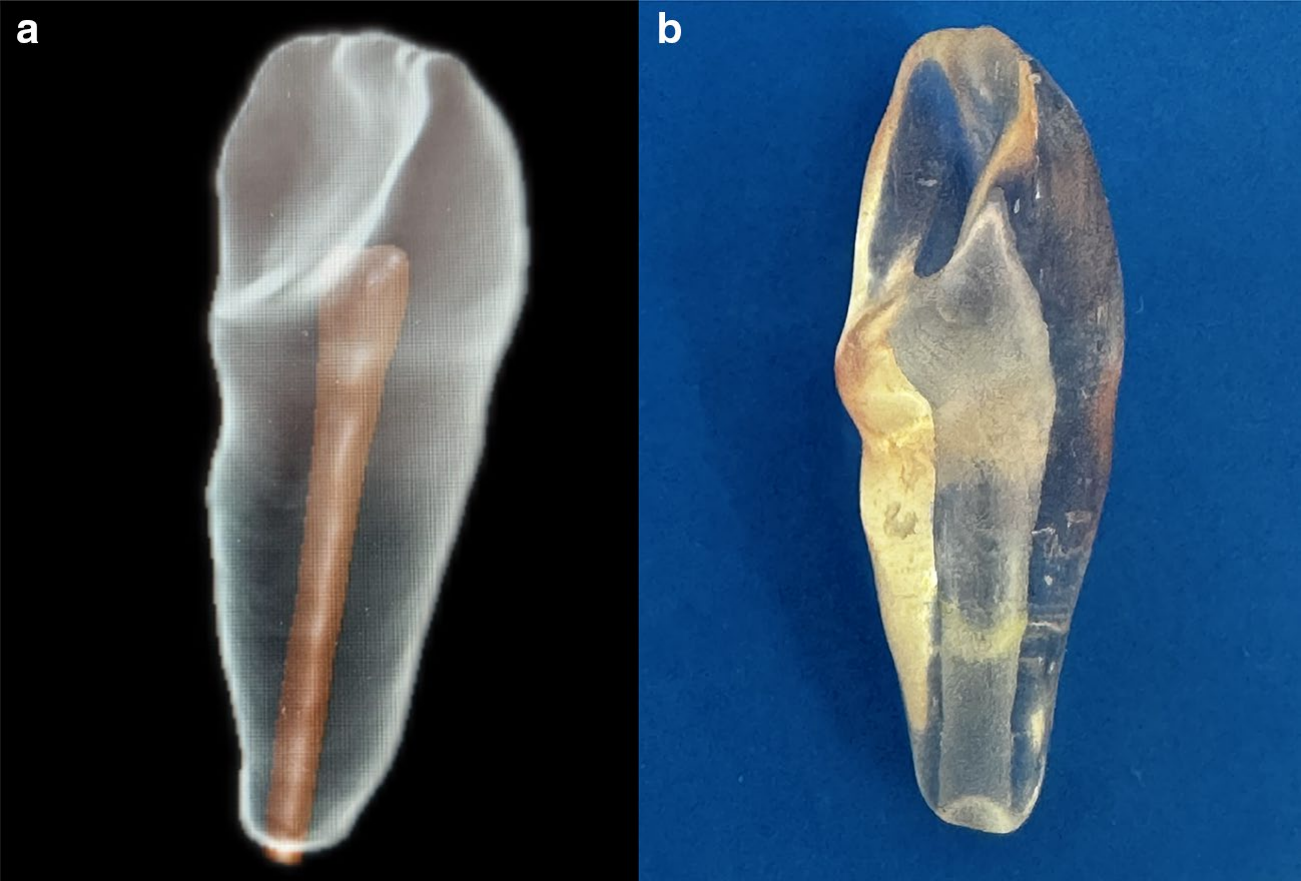
**a** The design stage of 3D-printed transparent tooth models via 3D design program (Meshmixer; San Rafael, CA, USA); **b** after the printing stage, the obtained 3D-transparent tooth model.

The methodology employed for the study was designed based on a similar study on apical extrusion [21]. The 3D-printed immature teeth models with permanent maxillary incisor morphology were created by modifying a previous study in the literature [22]. The 3D-printed transparent tooth models with a pulp chamber and open apex were specifically designed (Fig. 1a) and produced (Fig. 1b) in the dimensions we provided, by a manufacturer (EduDent Educational Materials, İstanbul, Turkey). The specific parameters of the resin tooth model were as follows: (i) a crown length of 6 mm and diameter of 5 mm (a conical coronal reservoir simulating an access cavity), (ii) a root length of 16 mm, (iii) a taper of 0.2 mm, and (iv) an apical diameter of 1.5 mm. The model was designed using a 3D design program (Meshmixer; San Rafael, CA, USA) and saved in the *.stl* format, afterward transferred it to a 3D printer (Anycubic Photon M3 Max, London, US). Consequently, we obtained 49 replicas with a resolution of 6.480 × 3.600 pixels. A transparent urethane–acrylate‐based photosensitive resin was used as the 3D printing material.

The teeth were fixed to the plastic lid of the transparent plastic tubes (Labsarf A.Ş, İstanbul, Turkey) with dimensions of 65 × 85mm, using cyanoacrylate glue (Henkel, Itapevi, SP, Brazil). Gutta-percha was inserted in each canal at the WL to plug the apex and prevent the agarose gel from flowing into the canals. The tubes were filled with 0.2% agarose gel (Difco Laboratories, Sparks, MD; pH = 7.3–7.4) including 1 mL of 0.1% m-Cresol purple (Sigma-Aldrich, St Louis, MO), which changes color from yellow (pH = 7.4) to purple (pH = 9) based on pH levels. The agarose gel changed color to purple upon the extrusion of NaOCl (pH = 11.4). After the gel had hardened, the lids were shut, and the teeth got stuck in the gel. All experimental procedures were completed within 2 h.

### Irrigation protocol

A rubber dam was placed on all sample teeth before starting the irrigation procedure to prevent the operator from seeing any color changes in the agarose gel. This ensures objectivity in the evaluation of the results. One experienced endodontist (V.A) performed all procedures. Subsequently, the specimens were randomly assigned to seven experimental groups (*n* = 7) based on the irrigation activation methods and insertion depths as follows: CSI-1, CSI-2, EDDY-1, EDDY-2, XP-1, XP-2, and SWEEPS.

#### Conventional syringe irrigation (CSI)

A syringe with a 30-gauge side-vented needle (Ultradent, South Jordan, UT, USA) was used to irrigate the canals. A total of 20 mL of 1% NaOCl were used for eight cycles. A stopper was inserted on the needle for WL control.

Group CSI-1 (*n* = 7): On specimens in group CSI-1, the needle was placed 1 mm shorter than the WL and used with a vertical motion.

Group CSI-2 (*n* = 7): On specimens in group CSI-2, the needle was positioned 2 mm shorter than the WL and used with a vertical motion.

#### Irrigation activation with EDDY

The canals were injected with NaOCI using a syringe, followed by sonic irrigation using an EDDY tip inserted into an air scaler (Micron, Tokyo, Japan). Eight cycles of irrigation were performed with a total of 20 mL of 1% NaOCI.

Group EDDY-1 (*n* = 7): The EDDY tip was placed 1 mm shorter than the WL and employed with a pecking motion.

Group EDDY-2 (*n* = 7): The EDDY tip was placed 2 mm shorter than the WL and employed with a pecking motion.

#### Irrigation activation with XP-endo Finisher file (XP-F)

The XP-endo Finisher file was inserted into a torque-controlled endodontic motor (VDW Gold, Munich, Germany). As recommended by the manufacturer, XP-F was inserted into the canal and activated at a speed of 800 rpm and 1 N/cm of torque with 7–8 mm upward and downward movements after reaching the WL. The irrigation solution inside the canal was refreshed continuously in eight cycles with a total of 20 mL of 1% NaOCl.

Group XP-1 (*n* = 7): The XP-F was inserted 1mm shorter than the WL and used with vertical movement.

Group XP-2 (*n* = 7): The XP-F was inserted 2 mm shorter than the WL and used with vertical movement.

#### Irrigation activation with SWEEPS

**Fig. 2:**
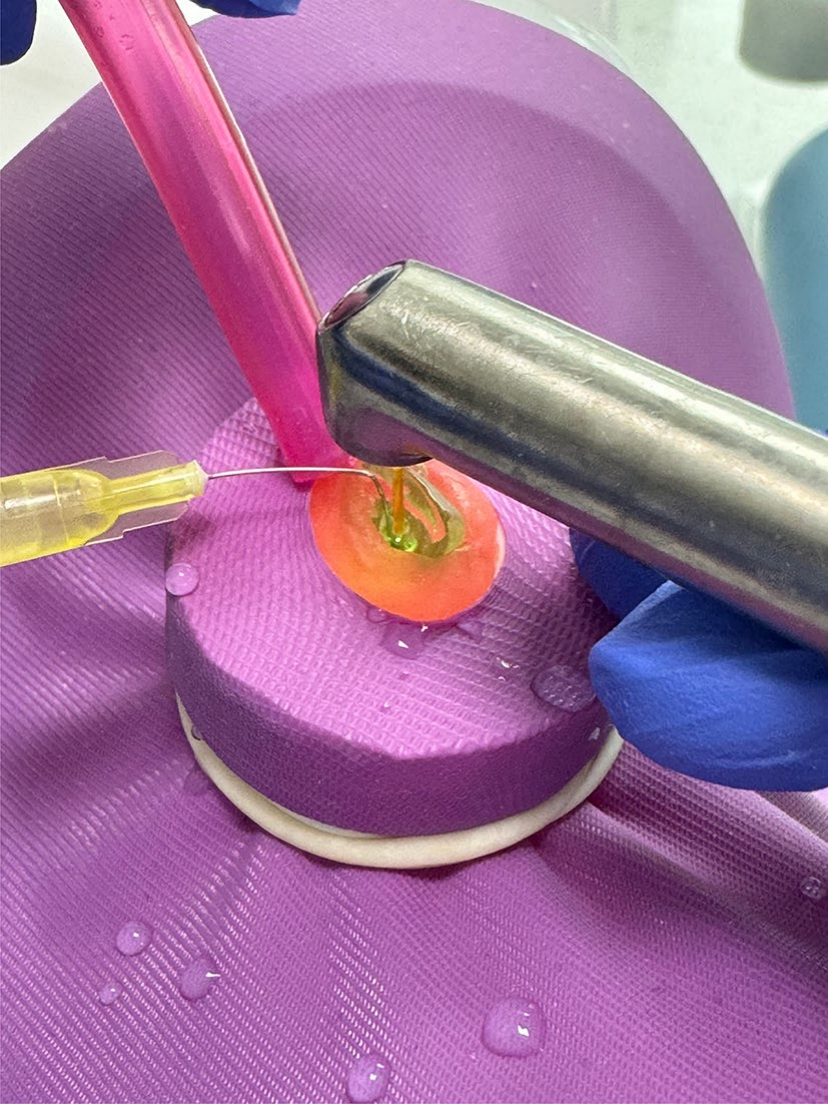
Irrigation activation with SWEEPS

For irrigation in the SWEEPS group (*n* = 7), Er:YAG laser device (LightWalker, Fotona, Ljubljana, Slovenia) with a wavelength of 2940 nm was used, employing the auto SWEEPS mode with 20 mJ energy per pulse, 15 Hz frequency and 50 µs time. The SWEEPS tip was inserted into in the access cavity and maintained in a stable position according to the manufacturer's instructions (Fig. 2). During the activation process, the irrigation solution inside the canal was refreshed continuously in eight cycles with a total of 20 mL of 1% NaOCl.

### Evaluation of apical extrusion

**Fig. 3:**
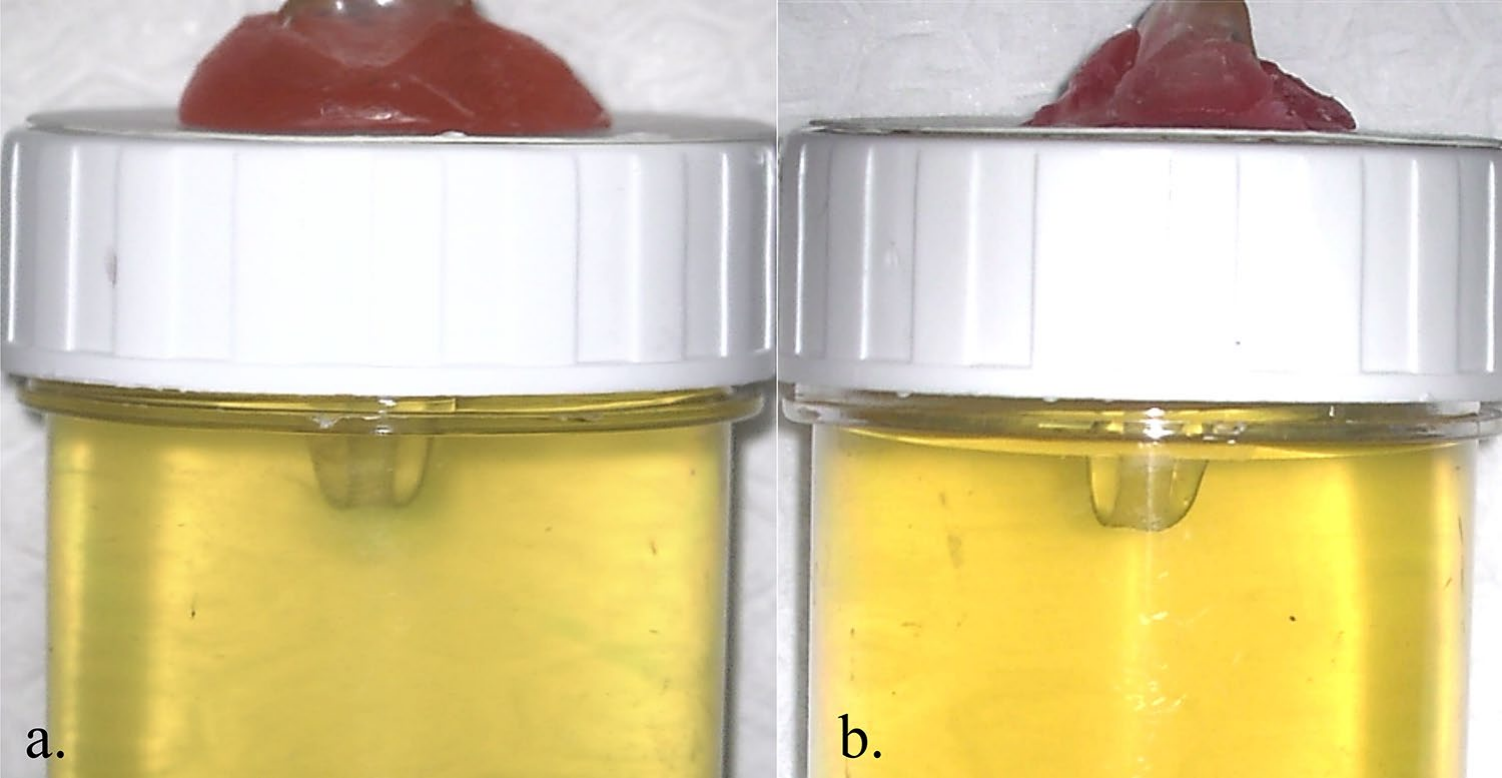
A view of bukkolingual direction (**a**) and mesiodistal direction (**b**) of a sample after completion of irrigation procedure

**Fig. 4:**
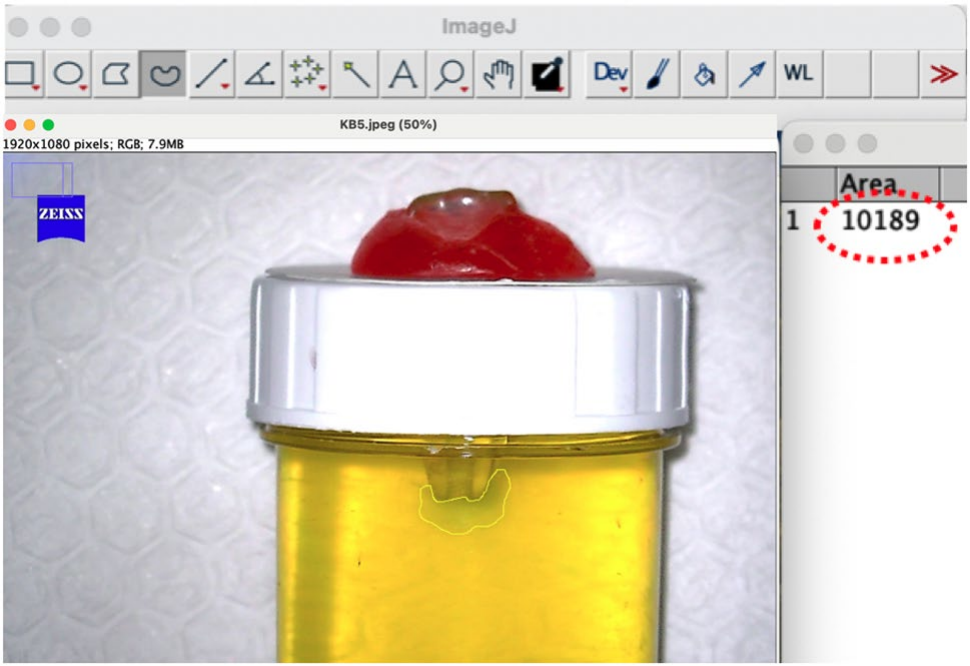
Analysis of a digital photograph to determine the area of color change using the ImageJ program

The tube was placed against a white background and photographed digitally from buccal/lingual and mesial/distal perspectives (Fig. 3), using a fixed distance (20 cm) digital camera. To assess the extrusion of NaOCl into the gel each sample was photographed and visually inspected. To standardize the dye diffusion time, the samples were photographed exactly 3 min after the completion of the irrigation activation procedure. The photos were analyzed using ImageJ program (NIH, MD, USA) to calculate the area of color change in pixels (Fig. 4). The ImageJ program automatically counted the pixels of irrigant extrusion area in each photograph, and the data were recorded by an examiner who was unaware of the experimental groups. The total number of pixels in each photograph was 1920 × 1080 pixels. The percentage of pixels indicating irrigant extrusion in each image was determined.

### Statistical analysis

Shapiro–Wilk test yielded values with *P* < 0.05 and the skewness and kurtosis values derived from their standard errors did not fall within the ± 1.96 threshold range, indicating a deviation from the normal distribution. To examine potential significant differences between the continuous variables, non-parametric tests, specifically Mann–Whitney *U* test and Kruskal–Wallis H test, were applied. Statistical significance was indicated by a significance level of *P* < 0.05.

## Results

The study was conducted without any reduction in the number of teeth samples. The study concludes that the area of apical extrusion caused by EDDY was significantly greater when the WL distance was 1 mm compared to 2 mm. (*Z* = 1.981, *P* = 0.048) (Table 1). It is worth noting that a significant difference was observed among the methods when working 1 mm (KW = 22.989, *P* < 0.001) and 2 mm (KW = 21.823, *P* < 0.001) short of the WL. Dunn's post hoc analysis in Table 2 revealed that the SWEEPS method had higher apical extrusion scores than EDDY at a working length distance of 2 mm and CSI at both working lengths.

**Table 1:** Comparison of the areas of apical extrusion in pixel percentages according to working length distances

Variables		n	Pixel (%)		Z	*P*
			Median (IQR)	Mean ± SD		
	CSI-1	7	0.202 (0.08)	0.198 ± 0.040	− 1.342	0.180
	CSI-2	7	0.015 (0.26)	0.128 ± 0.211		
	XP-1	7	0.445 (0.31)	0.512 ± 0.165	− 0.447	0.655
	XP-2	7	0.547 (0.20)	0.531 ± 0.115		
	EDDY-1	7	0.449 (0.09)	0.425 ± 0.072	− 1.981	**0.048**
	EDDY-2	7	0.377 (0.09)	0.360 ± 0.042		

Mann–Whitney *U* test (*P* < 0.05)

Significant values are in bold

**Table 2:** Comparison of apical extrusion areas in pixel percentages for equal working length groups

Variables		*n*	Pixel (%)		KW^*^	*P*	Post hoc^**^
			Median (IQR)	Mean ± SD			
	1) CSI-1^a^	7	0.202 (0.079)	0.198 ± 0.040	22.989	** < 0.001**	**1 < 4**
	2) XP-1^a^^b^	7	0.445 (0.307)	0.512 ± 0.165			
	3) EDDY-1^a^^b^	7	0.449 (0.087)	0.425 ± 0.072			
	4) SWEEPS^b^	7	0.973 (0.216)	0.979 ± 0.131			
	1) CSI-2^x^	7	0.015 (0.26)	0.128 ± 0.211	21.823	** < 0.001**	**1 < 4** **3 < 4**
	2) XP-2^ x^^y^	7	0.547 (0.20)	0.531 ± 0.115			
	3) EDDY-2^x^	7	0.377 (0.09)	0.360 ± 0.042			
	4) SWEEPS^y^	7	0.973 (0.216)	0.979 ± 0.131			

*Kruskal–Wallis (KW)-*H* test (*P* < 0.05)

**Different superscript letters in each column indicate significant differences between the groups according to post hoc test (*P* < 0.05)

Significant values are in bold

## Discussion

Upon examination of studies focusing on apical extrusion, it becomes evident that there is considerable variation in methodology and study design [23]. A significant number of studies have employed extracted teeth as experimental specimens in the analysis of apical extrusion, as they more accurately reflect clinical circumstances [24–26]. Nevertheless, it is challenging to achieve reliable dimensional and morphological standardization when utilizing natural teeth. In the majority of studies [27, 28], the crowns of the teeth were decoronated at the cementoenamel junction to achieve specific root lengths for standardization purposes. However, it has been reported that the crown acts as a reservoir for root canal irrigants, particularly when employing activation techniques [27]. The presence of crown may enhance fluid dynamics during activation and the possibility of advanced delivery of irrigants to the apical third [28], which could result in a change in the amount of apical extrusion [29]. In conclusion, one potential disadvantage of studying irrigation methods is the difficulty in selecting natural teeth with robust crowns, standardized root canals, and strict measures in the apical region [23].

The utilization of 3D-printed tooth models, which were employed in numerous research studies [30, 31], presents a notable advantage in that it allows for the standardization of dimensions of root canals, thus facilitating the comparison of the different irrigation techniques in a more reproducible manner than is possible with the extracted teeth [30]. Furthermore, the capacity to produce teeth with open apex in varying sizes and shapes may represent a notable advantage, particularly in the studies on regenerative endodontics.

The utilization of 3D-printed teeth, comprising a robust crown structure that serves as reservoirs for irrigation solutions, has yielded more realistic results in extrusion studies than natural teeth that have undergone decoronation [23]. One potential limitation of studies utilizing acrylic models is the possibility that the heat generated by rotary instruments may soften the resin material, thereby compromising the reliability of the experimental system [32, 33]. Nevertheless, during the design phase, the teeth were designed with an open apex and shaped to the required width, and then produced in accordance with these parameters. This eliminated the necessity for instrumentation during the experimental process, thus overcoming the aforementioned limitation.

Advancements in irrigation activation technologies may lead to a decreasing trend in the required apical preparation width for root canal therapy. This belief may be true for mature teeth but is unacceptable for immature teeth with a large apical diameter. Hence, the risk of apical extrusion during irrigation becomes more pronounced in immature teeth, posing a challenge for clinicians. Most studies on extrusion have been conducted on permanent teeth with mature apices [34–40], with limited experiments performed on immature or open apex teeth. Currently, limited studies have examined the ER:YAG laser methods on apical extrusion in teeth with immature apex [41, 42]. However, this is the first study to demonstrate the effect of using novel SWEEPS technology on apical extrusion of NaOCI in immature teeth.

Several studies have failed to include the potential impact of periapical tissues when assessing the extent of extrusion into air-filled vials [40, 43, 44]. This technique leads to an unrealistic scenario where there is no resistance from periapical tissue. Prior research has recognized this problem as a factor that leads to an exaggerated estimation of irrigant extrusion [23]. The present study used an agar gel model with a concentration of 1.5%, which has a density of 1045 kg/m3. This density is within the range of human periapical tissues, which typically vary between 1000 and 1100 kg/m^3^. Additionally, using a certified contrast solution helps replicate clinical conditions [23].

The amount of apical debris extruded increased as the level of irrigation technique approached the working length [37]. According to our findings, although there was a tendency to extrude more irrigant as the closer to working length; for XP-F and CSI groups, there was not a significant difference among the methods when performing 1 mm and 2 mm short of the WL, thus rejecting the first null hypothesis. The current study's findings are consistent with a study [45], which showed that irrigant extrusion increases as the apical diameter increases, regardless of needle insertion depth.

A novel introduced SWEEPS is an Er: YAG laser model that has been specifically designed to enhance the cleaning and disinfection efficacy of the photon-induced-photoacoustic-streaming (PIPS) technique. By providing two subsequent laser pulses to the irrigation solution within a certain period of time, the bubbles generated by the laser collapse more quickly, allowing the photoacoustic shock wave to pass through narrower root canals [46]. Shock waves pass through the irrigation solution and engage with the surrounding tissue and root canal wall, penetrating deep into the accessory canals to remove debris and microorganisms [47]. The increased pressure within the root canal can facilitate the flow of irrigants toward the apical area, potentially leading to the extrusion of irrigants [34, 48]. The study's results clearly demonstrate that SWEEPS causes a significantly higher degree of apical extrusion when compared to CSI. This phenomenon can be confidently attributed to the shock waves generated by the SWEEPS method.

It is impossible to compare specifically this result as there is no other study in the literature evaluating the effect of SWEEPS on apical extrusion in immature teeth. In a study evaluated the effect of SWEEPS on apical extrusion in teeth with closed apices, it was found that syringe irrigation caused more apical extrusion compared to the SWEEPS method [35]. Variations in apical diameters could be the reason for the discrepancy between their and our results. One of the limited studies examining the effectiveness of the SWEEPS method in clinical use is related to postoperative pain [49]. Evidence of a direct relationship between postoperative pain and apical extrusion has been demonstrated in the previous studies [49–51]. It was reported that PIPS and SWEEPS approaches were more successful than EDDY and PUI in avoiding and decreasing postoperative pain [49]. However, when it comes to apical extrusion, our findings yielded opposite results. While the main cause of postoperative pain is apical extrusion of both debris and irrigation solutions, we focused solely on NaOCI extrusion after completion of irrigation procedures.

Concerns regarding the irrigation activation methods in immature teeth are still a reality in endodontic practice. Considering that preserving the viability and proliferation potential of stem cells should be a primary concern of RET. Although the SWEEPS may appear to be a more advantageous irrigation method for RET due to its requirement for a smaller access cavity and avoidance of contact with the canal walls, it is noteworthy that this method resulted in more apical extrusion compared to EDDY at a working length distance of 2 mm, and CSI at both working lengths, thus rejecting the second null hypothesis.

The current study was conducted under laboratory conditions, which may not reflect real-life situations where factors such as gravity and pressure from surrounding tissues could influence the results. This may be considered as a limitation of the current study. The model demonstrated a matching of the canal geometry following root canal preparation at a specified size, which is suitable for irrigation and activation. However, the root canal system did not fully correspond to the actual anatomy of a natural tooth, which has apical delta, lateral canals, anastomoses, and other characteristics. This may be considered a second limitation of the present study.

It is important to note that each approach and methodology presented and reviewed in the current research has its own limitations. Consequently, there is currently no ideal methodology that fully meets the parameters of an acceptable apical extrusion study [23]. However, the novel approach of utilizing 3D-printed models that simulate the morphology of natural teeth incorporates considerations such as the standardization of apical dimensions and shape [23]. This allows for the attainment of more reproducible results when compared to studies utilizing extracted teeth [23]. On the other hand, it appears that the introduction of new and efficient irrigation activation techniques is about to happen soon. To thoroughly investigate the efficacy of novel irrigation activation techniques on apical extrusion, further research is imperative. Future studies should encompass a multitude of variables and strive for enhanced standardization, utilizing 3D-printed teeth that closely resemble natural teeth including features, such as the isthmus, lateral canal, apical delta, curved canal, or multiple canals.

## Conclusion

Clinicians should carefully interpret these findings to select the most effective irrigation strategy during RET. Due to the fact that, this study highlights the importance of selecting an appropriate activation method and activation tip positioned at the correct level to prevent apical extrusion. Furthermore, additional research is required to investigate irrigation activation techniques that not only improve cleaning effectiveness but also minimize apical extrusion and preserve apical tissues in immature teeth during RET.

## Data Availability

The datasets generated and/or analyzed during the current study are available from the corresponding author on reasonable request.
